# Composite Transport Model and Water and Solute Transport across Plant Roots: An Update

**DOI:** 10.3389/fpls.2018.00193

**Published:** 2018-02-16

**Authors:** Yangmin X. Kim, Kosala Ranathunge, Seulbi Lee, Yejin Lee, Deogbae Lee, Jwakyung Sung

**Affiliations:** ^1^Division of Soil and Fertilizer, National Institute of Agricultural Sciences, Rural Development Administration, Wanju, South Korea; ^2^School of Biological Sciences, The University of Western Australia, Perth, WA, Australia

**Keywords:** apoplastic barrier, aquaporins, composite transport model, exodermis, water and solute transport

## Abstract

The present review examines recent experimental findings in root transport phenomena in terms of the composite transport model (CTM). It has been a well-accepted conceptual model to explain the complex water and solute flows across the root that has been related to the composite anatomical structure. There are three parallel pathways involved in the transport of water and solutes in roots – apoplast, symplast, and transcellular paths. The role of aquaporins (AQPs), which facilitate water flows through the transcellular path, and root apoplast is examined in terms of the CTM. The contribution of the plasma membrane bound AQPs for the overall water transport in the whole plant level was varying depending on the plant species, age of roots with varying developmental stages of apoplastic barriers, and driving forces (hydrostatic vs. osmotic). Many studies have demonstrated that the apoplastic barriers, such as Casparian bands in the primary anticlinal walls and suberin lamellae in the secondary cell walls, in the endo- and exodermis are not perfect barriers and unable to completely block the transport of water and some solute transport into the stele. Recent research on water and solute transport of roots with and without exodermis triggered the importance of the extension of conventional CTM adding resistances that arrange in series (epidermis, exodermis, mid-cortex, endodermis, and pericycle). The extension of the model may answer current questions about the applicability of CTM for composite water and solute transport of roots that contain complex anatomical structures with heterogeneous cell layers.

## Introduction

Water and solutes, taken up by plant roots, use different pathways or routes, such as apoplastic and cell-to-cell (symplastic and transmembrane), to transport them into the vascular tissue in the stele (Steudle and Peterson, 1998; Steudle, 2000a; Ranathunge et al., 2017). This radial transport across the root has been explained by a “composite transport model (CTM),” which was firstly proposed by Steudle and his colleagues in 1990s (Steudle et al., 1993; Steudle, 1994; Steudle and Peterson, 1998). The CTM is directly related to the composite structure of the root. Different driving forces, such as hydrostatic/bulk and osmotic are used by different pathways. Many studies have shown that the usual driving force across roots is the tension, which is created by transpiration from the shoot and propagated into the root xylem (Steudle, 1995; Tyree, 1997; Steudle, 2000b, 2001). Therefore, the driving force across the root is a hydrostatic pressure gradient. However, in the absence of transpiration, the active pumping of nutrient ions into the xylem causes an osmotic water flow and a build-up of root pressure, in which the roots act as osmometers rather than just hydraulic resistors (Steudle, 2000b). During the osmotic water flow, in which water moves through the cell-to-cell pathway, it is likely that the contribution of the apoplastic pathway for water movement is negligible. The solutes are distributed throughout the porous apoplast, and it results in no osmotic pressure gradient along the apoplast. In contrast, during day time when transpiration is on, it results in the development of hydrostatic pressure gradient across the root that induces water flow both through the apoplastic and cell-to-cell paths. In this review, we examine how well the recent experimental results of water and solute transport across roots fit into the CTM. Recent studies also suggested that the conventional CTM consisting of parallel radial pathways shall be extended into a model with serial radial pathways (Meyer et al., 2011; Ranathunge et al., 2017).

## Composite Transport Model (CTM) of the Root

Before the proposal of CTM, roots were considered as perfect osmometers, explained by a semipermeable “single-equivalent-membrane model” (Steudle, 1994). This simple model did not consider and include the important phenomena such as variable root hydraulic conductivity (*Lp*_r_) and differences between hydrostatic/bulk and osmotic water flows. Over 60 years before the CTM was proposed, there was no convincing experimental evidence for the composite water and solute transport of roots. This model was proposed on the basis of measured values of hydraulic conductivity and other transport properties (solute permeability coefficients and reflection coefficients) of individual root cortical cells and entire roots (Steudle and Peterson, 1998; Steudle, 2000b). This model also successfully explains the results of greater *Lp*_r_ with increasing water flow across roots (Steudle and Peterson, 1998). It could also explain the low root reflection coefficients, differences between osmotic and hydraulic water flow, and differences between woody and herbaceous plants (Steudle and Peterson, 1998). According to the model, there are two parallel pathways, cell-to-cell path and apoplastic path, which have a quite different passive “selectivity” (reflection coefficient). Reflection coefficient is close to unity for the semipermeable cell-to-cell path (σ_s_^CC^ ≈ 1). In contrast, the porous apoplastic path does not select between water and solutes, which results in a reflection coefficient of close to zero (σ_s_^APO^ ≈ 0). These two pathways interact with each other, and it results in a circulation of water and low reflection coefficients of roots (Steudle, 1993, 2000a,b). According to the model, during transpiration, which creates a hydrostatic pressure gradient throughout the plant, the hydraulic resistance of (inverse of hydraulic conductance) roots will be low (Steudle, 2000a,b). In addition, the plant water supply from the root can be adjusted according to the demands of the shoot. In the absence of a transpirational water demand from the shoot, i.e., in the night, there will be only an osmotic gradient present due to the active uptake of solutes by the root (Steudle, 2000b). However, this will result in a much smaller root *Lp*_r_ and water flow. The composite transport of roots provides some switching of water and solute flows between pathways and a “coarse regulation of water flow” across roots, which is favorable for the plant (Steudle and Peterson, 1998). This model is also based on the composite root structure consisting of parallel arrangement of apoplastic and cell-to-cell paths. The acceptance of the model was further promoted by experimental results of puncturing endodermis of corn roots (Steudle et al., 1993).

## Recent Experimental Findings

After the CTM has been developed, many experimental research works on water and solute transport across the root were conducted and they have been explained in terms of the CTM (Knipfer et al., 2011; Hachez et al., 2012; Fricke et al., 2013; Suku et al., 2014; Vandeleur et al., 2014). However, recent experimental findings also raised concerns and debates about the validity of the CTM and some revisions and improvements to the model have been proposed (Knipfer and Fricke, 2010, 2011; Meyer et al., 2011; Gambetta et al., 2013; Ranathunge et al., 2017). Roots have complex anatomical structures with different cell layers in series and their permeabilities are different due to their apoplastic modifications, i.e., endo- and exodermis modify their cell walls by deposition of Casparian bands, suberin lamellae, and in some cases tertiary cell walls in the endodermis. Ranathunge et al. (2017) suggested to extend the CTM by adding components arranged in a series of the root (epidermis, exodermis, mid-cortex, and endodermis) in addition to the currently included components arranged in parallel (apoplastic and cell-to-cell pathways; see the section “Proposal to Improve the CTM: An Update”). Recent studies on cell-to-cell path in the root water transport are related to aquaporins (AQPs), and their expression patterns, which can be used as useful parameters to predict the role of AQPs in root water transport (Knipfer et al., 2011; Hachez et al., 2012; Vandeleur et al., 2014; Maurel et al., 2015).

## Role of Aquaporins

After the discovery of aquaporins (AQPs), their role in plant water transport in the single cell level has received a greater attention, and it has been questioned as to how much change in cell level impacts the change in the whole plant level. Inhibition of AQP function has been used to estimate the contribution of cell-to-cell path for the overall water transport, namely the AQPs based cell-to-cell path vs. apoplastic path (Maurel et al., 2015). When AQP function is inhibited, it has been shown that the hydraulic conductivity of the cells (*Lp*) and overall hydraulic conductivity of roots (*Lp*_r_) decreases (Lee et al., 2005a; Ye and Steudle, 2006; Knipfer et al., 2011). When Ye and Steudle (2006) inhibited the AQPs of maize roots by hydroxyl radicals, the fold change in cell *Lp* was by a factor of 9. However, in contrast, this reduction at the whole root level (*Lp*_r_) was threefold. The marked differences of fold changes of *Lp* in the cell level and root level were in line with the CTM. In addition to cell-to-cell path, it also agrees that the apoplastic path markedly contributes to the overall water transport across the roots. The exposure of cucumber to low temperature resulted in decreasing *Lp*_r_ and it was found to be related to the function of AQPs in the roots (**Table 1**; Lee et al., 2004, 2005a). When cucumber roots were exposed to low temperature, cell *Lp* decreased by a factor of as large as 16, and this magnitude of change was too big to be explained by viscosity change of water; so the authors suggested that the massive reduction of *Lp* was due to the inhibition of AQP function (Lee et al., 2005a). This finding was further supported by the experiment which involved in inhibition of AQPs by low temperature and mechanical stress (Lee et al., 2005b). This inhibition of AQPs at cell level by exposure to low temperature also had an impact on reduction of the *Lp*_r_ of cucumber roots. Here, root *Lp*_r_ decreased by a factor of 24 and it was an effect by both AQPs and root anatomy (Lee et al., 2005a). Knipfer et al. (2011) demonstrated that *Lp*_r_ of seminal root, adventitious root, and entire root system of barley can be reduced up to 40–74% by HgCl_2_ treatment, which inhibited AQP function. In the cell level, this treatment effect was greater and it reduced the *Lp* of cortical cells by 83–95%.

**Table 1:** Root hydraulic conductivity (*Lp*_r_), solute permeability (*P*_sr_), and reflection coefficient (*σ*_sr_) of different plant species, measured with different techniques.

Species	Root hydraulic conductivity *Lp*_r_ × 10^8^ (ms^-1^ MPa^-1^)		Root solute permeability *P*_sr_ × 10^9^ (ms^-1^)	Root reflection coefficient *σ*_sr_ (1)	Techniques	Reference
	Hydrostatic	Osmotic				
*Cucumis sativus* Whole root system	6.4–7.9 (25°C) 2.7–7.9 (13°C)	1.2–2.4 (25°C) 0.2–0.8 (13°C)			Root pressure probe	Lee et al., 2004
	
	12.2 (20°C) Cell *Lp*:160	3.2 (20°C)			Pressure chamber and osmotic flow Cell pressure probe	Lee et al., 2005a

*Vitis berlandieri* × *Vitis rupestris* Fine root (1) Root tip (2) Secondary growth portion	50 10	0.4 0.02			Pressure chamber and osmotic flow	Gambetta et al., 2013

*Hordeum vulgare* Seminal root (1) Root medium circulating (2) Root medium stagnant	12.2 3.2	5.1 0.4		NaCl: 0.7 NaCl: 0.4	Root pressure probe	Knipfer and Fricke, 2010

*H. vulgare* Seminal root end-segment (1) Root medium circulating (2) Root medium stagnant	9.4 9.7	9.5 4.2	Ethanol: 12.5 NaCl: 2.8 KCl: 2.5 Mannitol: 1.7 Sucrose: n.m. K_4_[Fe(CN)_6_]: n.m.	Ethanol: 0.35 NaCl: 0.69 KCl: 0.68 Mannitol: 0.90 Sucrose: 0.45 (non-corrected) K_4_[Fe(CN)_6_]: 0.61 (non-corrected)	Root pressure probe	Ranathunge et al., 2017

Besides inhibiting AQP function, the contribution of AQPs for the overall hydraulic conductivity of roots was estimated by comparing the hydraulic conductivities measured by hydrostatic and osmotic forces (Steudle, 1993, 2000a; Ranathunge et al., 2004; Chaumont and Tyerman, 2014). In cucumber and figleaf gourd, the *Lp*_r_ measured by changing hydrostatic force (*Lp*_r_^Hy^) was larger by a factor of 3 than measured by changing osmotic force (*Lp*_r_^Os^; Lee et al., 2004, 2005a; **Table 1**). The substantial differences in hydrostatic and osmotic *Lp*_r_ have been explained by the CTM in terms of a preferred apoplastic water flow under hydrostatic pressure gradient. Fricke et al. (2013) showed that *Lp*_r_^Hy^ and *Lp*_r_^Os^ were in the same range for young wheat roots either with NaCl treatment or non-treatment (control), indicating a significant contribution of cell-to-cell path for the overall root water transport.

Depending on the age of the root, the contribution of AQPs for overall *Lp*_r_ was different (Gambetta et al., 2013). In grapevine, the hydrostatic *Lp*_r_ was 100-fold greater than the osmotic *Lp*_r_ in both the tip (younger zone) and the zone with a secondary growth, which can be explained in terms of a markedly greater contribution of the apoplastic path for the overall root *Lp*_r_ compared with the cell-to-cell path (**Table 1**). Once AQPs were inhibited by a treatment with H_2_O_2_, only the osmotic *Lp*_r_ of the tip zone was substantially reduced; however, in contrast, there was no significant reduction in *Lp*_r_ of the secondary growth zone, which indicates there was no impact of AQPs in the *Lp*_r_ of the latter zone (Gambetta et al., 2013).

Depending on the species, the role of AQPs can be different (Maggio and Joly, 1995; Bramley et al., 2009; Sutka et al., 2011). Bramley et al. (2009) compared the contribution of AQPs on root *Lp*_r_ of lupin and wheat roots. In wheat, once the AQPs were inhibited by heavy metals, the whole root system *Lp*_r_ decreased by a factor of 2. On the other hand, the inhibition of AQPs by heavy metals in lupin roots did not change the *Lp*_r_ of whole root system. Tomato and Arabidopsis showed predominant role of cell-to-cell path and it was 57% and up to 64% of *Lp*_r_, respectively (Maggio and Joly, 1995; Sutka et al., 2011; Maurel et al., 2015).

Composite transport model challenged the role of AQPs in water transport across the root during the transpiration (Chaumont and Tyerman, 2014). According to the CTM, most of water is transported through the apoplastic pathway when bulk flow of water occurs by transpiration. The negative pressure or tension, created by transpiration, directly propagates through the continuous apoplast of the plant. This does mean that the extent of water transport through the cell-to-cell path that is regulated by AQPs can be restricted during transpiration. This was also true for the secondary growth zone of grapevine fine roots (Gambetta et al., 2013). Steudle (2000b) expected that highly suberized roots would not allow much water flow across the apoplast; therefore, water flow across the cell-to-cell path (regulated by AQPs) should play a role (fine regulation of water uptake). However, Gambetta et al. (2013) did not see this for the secondary growth zone of grapevine fine roots, in which *Lp*_r_ was at least 10-fold smaller than root tip. In the secondary growth zone, the expression of AQP genes was lower than the younger zone and the inhibition of AQPs by H_2_O_2_ treatment did not reduce *Lp*_r_^Hy^ further. According to Gambetta et al. (2013), despite having low *Lp*_r_, even the suberized roots were taking up significant amount of water through the apoplast. In addition, woody plants including grapevine have much smaller *Lp*_r_^Os^ than *Lp*_r_^Hy^, and it is smaller by a factor of 100. It concludes that the apoplastic pathway dominates for the overall water transport in woody roots.

Overall, the role or contribution of AQPs in root water transport is variable depending on the age of roots with varying development of apoplastic barriers, plant species, and driving forces (hydrostatic and osmotic).

## Role of the Root Apoplast

The extra-cellular matrix of the walls around most living cells is porous, the pores being water-filled in all but very exceptional circumstances (Münch, 1930). The apoplast was considered as a physical continuum through which water and solutes can freely move either by bulk flow in the presence of a transpirational force, where solutes can be dragged by water or by simple diffusion in the absence of transpiration. It has been documented that the transport of water and solutes can be reduced by the apoplast in which cell walls are impregnated with or deposited on the cell walls of non-permeable substances such as suberin and lignin, as for example in the endodermis and exodermis of roots (Steudle and Peterson, 1998; Steudle and Ranathunge, 2007; Ranathunge et al., 2011). Nevertheless, there are some studies documented that even in the presence of apoplastic barriers in the cell walls (e.g., the Casparian band and suberin lamellae) the apoplast is more permeable than previously anticipated (Clarkson et al., 1987; Ranathunge et al., 2004; Steudle and Ranathunge, 2007). Moreover, Schreiber et al. (2007) also concluded that suberization of cell walls does not necessarily result in complete impermeability of the apoplast to water or solute transport. However, the extent of suberization of the endo- and exodermis is highly variable, and depends on both species and environmental conditions.

Although cell walls are demonstrated as imperfect barriers for water in some experiments, several studies favor the view that suberin and lignin act as virtually impermeable barriers for ions, gases, and pathogens (Lux et al., 2004; Armstrong and Armstrong, 2005; Ranathunge et al., 2008, 2011; Meyer et al., 2011; Ranathunge and Schreiber, 2011; Kotula et al., 2014). For example, Arabidopsis-enhanced suberin mutant (*esb1*) with twice the suberin content of wild type had significantly lower content of nutrient ions in the shoot due to reduced nutrient uptake of roots (Baxter et al., 2009). In rice, induced suberin in the endo- and exodermis as well as elevated lignin in sclerenchyma cells by stagnant growth markedly reduced NaCl permeability (Ranathunge et al., 2011) and radial oxygen loss (Kotula et al., 2009) of rice roots. Similarly, pre-exposure of rice plants to moderate salt stress resulted in increased suberin depositions and significant reduction in NaCl and water uptake of roots (Krishnamurthy et al., 2009, 2011). Further, when grown in higher than optimum level of (NH_4_)_2_SO_4_, a commonly used nitrogen fertilizer in rice fields, roots deposited significantly higher amounts of suberin, both in the endo- and exodermis (Ranathunge et al., 2016). This resulted in markedly lower uptake rates of (NH_4_)_2_SO_4_, KH_2_PO_4_, and NaCl in rice roots.

## Exodermis as a Transport Barrier in Roots

Many plant species form an exodermis (the outermost cortical layer with Casparian bands) in their roots (Perumalla et al., 1990; Peterson and Perumalla, 1990; Ma and Peterson, 2003). Casparian bands are located in the anticlinal walls, and developed by impregnation of the primary walls with lignin and suberin (Schreiber et al., 1999). A major role of a Casparian band is to block the apoplastic diffusion of ions, as well as occlude the solvent drag of ions into the stele during transpirational bulk flow of water. It makes the endodermis and exodermis to act as filters for ions through the apoplast and then the membranes become the control points. For instance, development of an exodermis in onion roots resulted in developing an impermeable barrier to Ca^2+^ ions (Cholewa and Peterson, 2004). In young corn roots, formation of an exodermis by mist culture decreased *Lp*_r_ by fourfold (Zimmermann and Steudle, 1998). In onion, an exodermis development during root maturation caused a substantial reduction in *Lp*_r_ (Melchior and Steudle, 1993). Meyer et al. (2011) measured *Lp*_r_ of *Iris germanica* roots with and without multiseriate exodermis. When measured using a pressure chamber, *Iris* roots with an exodermis were less permeable for water by a factor of 2 compared with *Iris* roots without exodermis. It demonstrated that exodermis provides a significant resistance to water flow. The measured *Lp*_r_ values using a root pressure probe and a pressure chamber were often somewhat different for the same plant species; in general, pressure chamber values were relatively smaller than root pressure probe values (Ranathunge et al., 2003, 2011). This finding requested to extend the current CTM with parallel resistances (apoplastic compared to cell-to-cell) into one with series resistances (endodermis compared to exodermis; see below).

## Challenges Related to CTM

### Key Question 1: Are Roots Perfect Osmometers? Can a Single Homogenous Membrane Model Be Applied for Root Transport?

Traditionally, roots have been viewed as nearly ideal osmometers comparable to a cell (reflection coefficient σ_sr_ = 1; solute permeability *P*_sr_ = 0; Weatherley, 1982). The endodermis with the fully developed Casparian band was considered as “the root membrane” and the osmometer model was called a “single-equivalent-membrane model” (Dainty, 1985). Steudle (1994) changed and improved “single-equivalent-membrane model” to “composite barrier model” including properties of roots such as leakage of solute (*P*_sr_ > 0) and deviation from the perfect osmometers (σ_sr_ < 0). Recently, Knipfer and Fricke (2010) claimed that the measured reflection coefficient of barley roots for NaCl was between 0.4 and 0.7 but the real reflection coefficient was very close to unity (**Table 1**). The authors concluded that the barley root behaves as a perfect osmometer. Ranathunge et al. (2017) measured the *Lp*_r_ of barley roots and they extended measurements of *P*_sr_ and σ_sr_ for various solutes (**Table 1**). The measured values of Ranathunge et al. (2017) suggested that the CTM should be extended by adding serial resistances across the roots.

In general, roots behave like an osmometer for certain solutes, but they do not behave similar to the “single homogeneous membrane model” described by Weatherley (1982). Instead, roots show complex behavior in terms of *P*_sr_ and σ_sr_ that are different from a single cell membrane model. For example, reflection coefficient of between 0.2 and 0.8 should refer to a very high solute permeability for a “single homogenous membrane,” but for roots, the root solute permeability can be low (Steudle and Peterson, 1998).

### Key Question 2: More Apoplastic Barrier *Per Se* vs. More AQPs?

Do deposition of stronger apoplastic barriers result in expressing more AQP genes along the root axis, in order to maintain higher water uptake rates? Gambetta et al. (2013) expected that there would be more AQPs expressed at the mature root zones where highly suberized strong apoplastic barriers were deposited in the roots of grapevine, because CTM proposed that AQPs play a role of fine tuning for water flow in older suberized parts, which lack a substantial apoplastic water flow (Steudle and Peterson, 1998). However, differently, Gambetta et al. (2013) observed more AQPs in the growth zone where there is weak or incomplete apoplastic barriers compared with the mature part. Similarly, Knipfer et al. (2011) also found that cortical cell *Lp* was smaller in the fully mature zone of the barley seminal root than in younger transition zone. It can be expected that the primary role of AQPs in the growing tissue is facilitating cell-level water relations. Alternative explanation for role of AQPs in the growing tissue of grapevine is that these roots can build a highly permeable young root zone for water while having less permeable mature root zone in order to take up water from the young part of root, similar to the leaky cable theory (Landsberg and Fowkes, 1978; Zwieniecki et al., 2003; Zarebanadkouki et al., 2013). According to this theory, tight barrier in the older part is needed to produce high water potential gradient between young root xylem collar and adjacent soil. This allows the young part of the root to take up water when it reaches available water while other older parts of the root are still in dry soil.

In terms of the radial transport of water, presence of more AQPs at certain suberized barriers might be partly correct. As proposed by Schäffner (1998), AQPs might be concentrated in the passage cells of the endodermis. Hachez et al. (2006) showed higher expression of some AQPs in the endodermis and exodermis of maize roots. They also showed that *Zm PIP2;5* and *Zm PIP1;2* protein levels increased in the exodermis and epidermis, when maize was grown in aeroponics that resulted in development of an exodermis with Casparian bands (Hachez et al., 2012). Ranathunge and Schreiber (2011) emphasized that suberin lamellae mask the plasmalemma in the endodermis and this results in reduced contribution of AQPs to the total water transport. In roots of Arabidopsis, stele had more AQPs and they allowed a greater water flux into the xylem vessels due to centripetal water transport (Postaire et al., 2010; Maurel et al., 2015).

### Key Question 3: Is Water Flow Solely through the Apoplast Enough to Overcome the Transpirational Demand by Shoot or Is There Any AQP Involvement?

It is a key question whether AQPs have a role in increasing *Lp*_r_ during the elevated transpiration or not (Maurel et al., 2016). Several recent studies correlated increased transpiration rate with root AQP expression (Almeida-Rodriguez et al., 2011; Sakurai-Ishikawa et al., 2011; Kuwagata et al., 2012; Laur and Hacke, 2013). There are cases in which AQPs play a role under a hydrostatic pressure gradient (Henzler et al., 1999; Vandeleur et al., 2009). According to Henzler et al. (1999) lotus root has a peak in *Lp*_r_^Hy^ and *Lp*_r_^Os^ during the day and the abundance of root AQP transcripts showed a similar pattern to the variation in root *Lp*_r_. When Vandeleur et al. (2009) used the whole root system of a grapevine with pressure gradient measurement, *Lp*_r_^Hy^ had a peak during the day and one AQP isoform, *VvPIP1;1* expression pattern matched to the *Lp*_r_^Hy^ variation pattern. On the other hand, the *Lp*_r_^Os^ of rice root system measured using an osmotic pressure gradient showed a peak in *Lp*_r_^Os^ during the day time (Sakurai-Ishikawa et al., 2011). In the case of rice root, the authors did not measure *Lp*_r_^Hy^ using hydrostatic pressure gradient to compare it with osmotic *Lp*_r_^Os^. Interestingly, they also showed that the higher AQP gene expression in response to transpiration increases separating from the light effect. Almeida-Rodriguez et al. (2011) showed that the *Lp*_r_^Hy^ of poplar root segment measured by a vacuum method was higher in the light-treated plants, which had been grown in the shade than in the non-treated plants. The transpiration increase induced by light also increased root AQP transcripts.

### Key Question 4: How Valid/Acceptable Are *Lp*_r_ Values Measured by Different Methods?

*Lp*_r_ values measured using a root pressure probe were different depending on the mode of measurements (Bramley et al., 2007). These authors measured higher root *Lp*_r_ using the hydrostatic relaxation with the root pressure probe than the pressure clamp with the root pressure probe. They concluded that the pressure clamp is the better method for measuring *Lp*_r_ as it gives a sufficient time for pressure propagation through the root, and there is no significant osmotic effect due to unstirred layers. Similarly, higher root *Lp*_r_ was obtained for corn roots by using hydrostatic relaxation with the root pressure probe than either by using hydrostatic relaxation after the pressure clamp or by the pressure clamp with the root pressure probe (Knipfer et al., 2007; Knipfer and Steudle, 2008). These authors explained these phenomena that there was an effect of unstirred layers to reduce the root *Lp*_r_ after the pressure clamp causing large quantities of water and solute flows across the root and solute polarization at the endodermis (convection vs. diffusion model; C/D model). Detailed computer simulation of water and solute flows could successfully reproduce experimental curves of pressure probe measurements. They concluded that pressure relaxation with the root pressure probe is the better technique to measure root *Lp*_r_ than the other methods that produce massive water flows across the roots to measure *Lp*_r_, such as pressure clamp with the root pressure probe.

Chaumont and Tyerman (2014) questioned whether the comparison of osmotic and hydrostatic *Lp*_r_ would be accurate to predict the contribution of cell-to-cell path for overall water transport. They also mentioned that a caution is necessary as pressure-driven hydrostatic water flow may influence the gating of AQPs in the plasma membrane. On the other hand, Meyer et al. (2011) suggested that the amount of water injected into the root xylem (exosmotic) during root pressure probe experiments might not be enough to measure the barriers of the whole roots, especially the exodermis of thick roots with many cortical cell layers, such as *I. germanica*.

Suku et al. (2014) indicated that usage of excised root system to measure *Lp*_r_ could be different from *Lp*_r_ of the intact transpiring plants where feedback from the shoot to root is involved. In favor of this idea, some experiments showed that transpiration and shoot topping affected root AQP activity (Almeida-Rodriguez et al., 2011; Sakurai-Ishikawa et al., 2011; Kuwagata et al., 2012; Laur and Hacke, 2013; Vandeleur et al., 2014).

Challenges in the *Lp*_r_ measurements made it difficult to build a general root hydraulic model. On the other hand, it revealed the complexity of the roots that depends on the complex anatomical structures and highlighted the requirement of a progress of general root hydraulic model.

## Proposal to Improve the CTM: An Update

**FIGURE 1:**
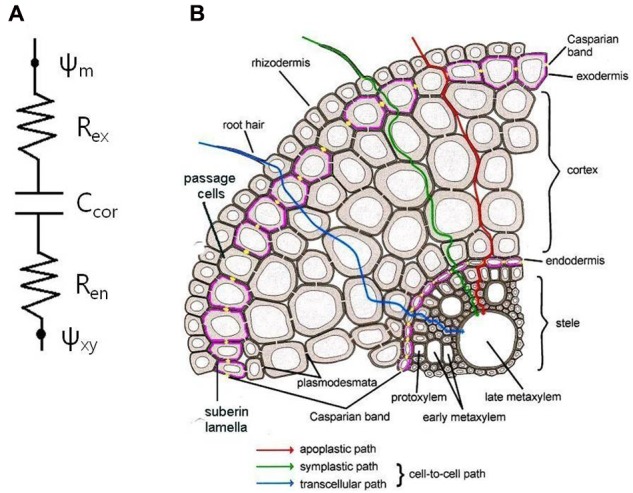
Extended composite transport model. **(A)** Electric analogy with series of resistance–capacitor–resistance. Ψ_m_, water potential in medium; Ψ_xy_, water potential in xylem; *R*_ex_, exodermis resistance; *R*_en_, endodermis resistance; *C*_cor_, cortex capacitance. **(B)** Schematic diagram of a root cross-section showing pathways of water and solute transport into the stele. Casparian bands (yellow dots) and suberin lamellae (pink lines) in the endo- and exodermis interrupt water and solute transport into the stele.

Current CTM consists of parallel arrangement of apoplastic and cell-to-cell paths. Considering roots with an exodermis, the concept of transport should be extended into series of resistance–capacitor–resistance (**Figure 1**). Here, the storage capacity of roots may give transient effects (Meyer et al., 2011). Even in the absence of an exodermis, the same phenomena would apply for the roots in soil, where the rhizosphere may act as another additional transport barrier similar to the exodermis, or even stronger. During drought stress, when the soil dries, an air gap would form in between the root surface and soil due to shrinkage of roots and soil, resulting in decrease of hydraulic conductivity of root–soil interface (Carminati et al., 2009).

## Conclusion

Since the development of the CTM by Steudle and colleagues, it has been employed to most of the studies in water and solute transport across roots. Although the CTM has been a well-accepted, conceptual model to explain the root transport phenomena, recent studies have raised some challenging and open questions regarding the contribution of AQPs for the total water flow and the presence of exodermis in roots and their relevance to the CTM. As mentioned earlier in the previous sections of this review, further expansion of the concept of CTM is necessary to provide answers and actual computation of this model into a computer simulation would assist further.

## Author Contributions

YK and JS conceived the study. YK, KR, and JS prepared the manuscript. All authors participated in the discussion.

## Conflict of Interest Statement

The authors declare that the research was conducted in the absence of any commercial or financial relationships that could be construed as a potential conflict of interest.
